# Three-Dimensional Evaluation of Condylar Position and Joint Spaces Following Orthodontic Treatment With Quadruple Premolar Extractions

**DOI:** 10.7759/cureus.67176

**Published:** 2024-08-19

**Authors:** Kaiyang Xue, Qingran Li, Sihan Yang, Yuxiang Zhao, Jiawei Li, Chenghao Zhang, Wen Liao

**Affiliations:** 1 Department of Orthodontics, State Key Laboratory of Oral Diseases and National Clinical Research Center for Oral Diseases, West China Hospital of Stomatology, Sichuan University, Chengdu, CHN

**Keywords:** premolar, orthodontic, tmj, fixed appliance, cbct

## Abstract

This study aimed to assess the alterations in the temporomandibular joint among adult patients undergoing orthodontic treatment involving the extraction of four premolars. A cohort of 44 adults, with a mean age of 24.2 years, underwent orthodontic therapy that included quadruple premolar extractions. Cone-beam computed tomography scans were performed before and after the treatment to evaluate the temporomandibular joints. The three-dimensional assessment focused on the condylar position relative to the cranial base and the articular fossa, the axial condylar rotation, and the joint spaces. Notably, a significant posterior shift of the condyle was detected (P≤0.01), averaging a 0.41mm retraction. The posterior joint space narrowed by 0.32mm post-treatment. Additionally, a medial tilt of 0.62° in the condyle’s long axis was observed in the frontal plane. No significant changes were recorded for the other condylar positions, rotations, or joint space dimensions. The findings suggest that orthodontic treatment with four premolar extractions may instigate condylar repositioning and rotation. These insights can inform refinements in treatment protocols.

## Introduction

Tooth extraction is a commonly employed technique in orthodontic treatment, aimed at achieving a balance between tooth and bone quantity by reducing the number of teeth. Its primary goal is to address issues such as crowded teeth, sagittal, transverse, and vertical discrepancies between the upper and lower dental arches, as well as potential malocclusions of the jaw relationship [1,2]. Clinicians often opt for the extraction of first and second premolars, followed by molar traction to close the resulting gaps [3]. In East Asia, where Mongolian populations exhibit more pronounced differences in alveolar bone-dental arch, the extraction rate is higher [4]. Scholars have conducted statistical analyses of patients' medical histories and identified a significant association between orthodontic treatment and temporomandibular joint disorders [5]. Some patients have reported experiencing temporomandibular joint or surrounding pain after undergoing tooth extraction orthodontic treatment [6]. Additionally, there have been cases of temporomandibular joint sounds following orthodontic treatment [7]. However, other studies have found that individuals with Class II malocclusion and muscle-origin temporomandibular joint disorder symptoms appear to benefit functionally from orthodontic treatment [8]. Moreover, the number of patients with joint sounds at the end of the orthodontic treatment phase is lower than before the treatment [9].

Existing evidence from clinical research indicates that functional orthodontic treatment leads to changes in the position and skeletal structure of the temporomandibular joint in the short term, as compared to untreated control groups [10]. Ali et al. discovered that premolar extraction may influence condylar position, as measured by the joint space index [11]. Koide et al. found that after extraction or non-extraction orthodontic treatment, the condyle protruded at a greater angle to the Frankfurt horizontal plane, and the total, upper, and lower heights of the joint cavity significantly increased. Additionally, the anterior-posterior widths of both sides of the joint cavity decreased significantly, indicating adaptive bone remodeling of the temporomandibular joint [12]. However, their study suffered from a high degree of sample heterogeneity, a small sample size, and heavy artifacts in the cone-beam computed tomography (CBCT) images, which could lead to image distortion. Kevin et al. observed that the condyle became more concentric and noted a significant reduction in the left, right, anterior, posterior, and upper joint spaces in the majority of subjects during orthodontic treatment [13]. However, their study did not analyze the impact of tooth extraction on the results and is still limited to two dimensions. Alhammadi et al. found a statistically significant posterior shift of the condyle relative to the vertical plane. They also observed an increase in the anterior joint space and a decrease in the posterior joint space after treatment [14]. However, their study only included patients undergoing extraction of the upper first premolar. Most existing studies on the temporomandibular joint after orthodontic tooth extraction treatment use MRI to evaluate the relationship of the disc. However, patients without temporomandibular joint disorders typically do not opt for MRI. Due to the lack of standardized methods, small sample sizes, and limited comprehensive assessments on a three-dimensional level, the quality of the existing evidence is low [10].

Our study is based on three-dimensional (3D) scanning and digital technology, utilizing CBCT's three-dimensional reconstruction and anatomical landmark overlap, and assisted by Mimics and Dolphin Imaging software. By quantitatively analyzing the aforementioned CBCT reference points and various indicators, this study assesses the impact of orthodontic tooth extraction on the temporomandibular joint in patients, providing orthodontists with valuable insights for clinical diagnosis, treatment, treatment planning, risk avoidance, and prognosis evaluation.

## Materials and methods

Subjects

This retrospective analysis focuses on orthodontic treatment conducted at the Department of Orthodontics, the West China Hospital of Stomatology, Sichuan University in Chengdu, China. The study period lasted from April 2016 to January 2023. Patient data was collected from the hospital's medical record database and carefully reviewed. The researchers examined the recorded diagnoses and treatment characteristics of the patients. The research samples were selected based on specific inclusion criteria, which are as follows.

Inclusion Criteria

Before and after orthodontic treatment, patients must have had CBCT images taken within two weeks. The CBCT images should cover the cranial and maxillofacial skeletal structures from the orbitals to the mandibular body, and the imaging data should be clear and free of artifacts. The patients must be older than 18 years and have all teeth from the central incisors to the second molars with no supernumerary tooth, tooth defect, or metallic restorations. The patients must have had four premolars extracted during orthodontic treatment. The patients must have used fixed appliances for their orthodontic treatment. Moderate anchorage should have been maintained during space closure. After treatment, the patients should have achieved complete space closure and good functional occlusion.

Exclusion Criteria

Patients with a history of para-functional habits or clinically diagnosed temporomandibular joint (TMJ) disorders, those who underwent previous TMJ surgery, and those with CBCT images that failed to cover both sides of the TMJ were excluded.

The process of obtaining medical history and conducting clinical examination was conducted in accordance with the Research Diagnostic Criteria for Temporomandibular Disorders (RDC/TMD). The utilization of this diagnostic system promotes consistency and the ability to reproduce accurate diagnoses and classifications of temporomandibular disorders (TMD).

The CBCT images in this study were acquired using a 3D Accuitomo CBCT machine (3D Accuitomo, Morita Group, Tokyo, Japan), which was configured based on the recommendations provided by the manufacturer (140 × 100 mm Field of View (FOV), 85 kV, 4.0 mA, and 360° rotation). The voxel size of the images was 125 µm. After acquisition, the CBCT data were saved in DICOM multifile format. All patients underwent treatment with the Damon Q self-ligating orthodontic appliance (Damon Q; Ormco Corporation, Brea, CA, USA).

Data preparation before measurement

**Figure 1:**
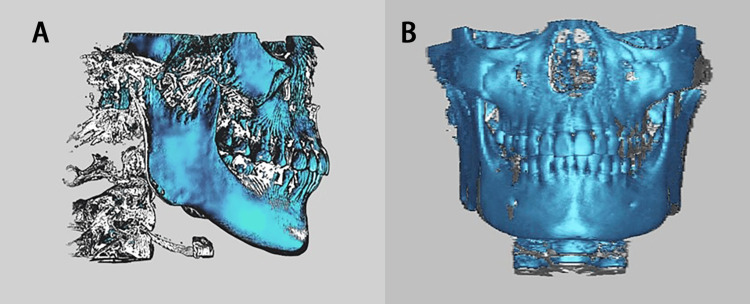
Landmark cone-beam computed tomography (CBCT) superimposition profiles The same four landmarks were included: ANS (the tip of the anterior nasal spine), PNS (the tip of the posterior nasal spine), OrL (the most inferior point of the left bony orbit), and OrR (the most inferior point of the right bony orbit). (A) Image overlap in the sagittal plane (B) Image overlap in the coronal plane

**Figure 2:**
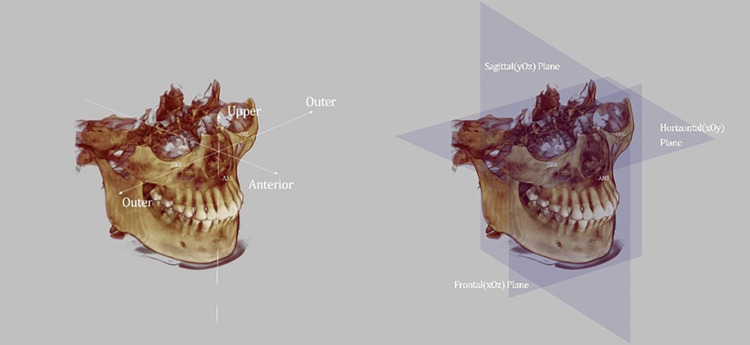
The maxilla-based coordinate system is established by utilizing four skeletal landmarks The maxilla-based coordinate system is established by utilizing four skeletal landmarks: ANS (the tip of the anterior nasal spine), PNS (the tip of the posterior nasal spine), OrL (the most inferior point of the left bony orbit), and OrR (the most inferior point of the right bony orbit). These landmarks form the foundation for the coordinate system. To establish the horizontal plane, a plane is created that passes through ANS and PNS, running parallel to the OrL-OrR line. The sagittal plane is defined as a plane that passes through ANS and PNS, perpendicular to the horizontal plane. Lastly, the frontal plane is defined as a plane that passes through ANS, perpendicular to both the horizontal and sagittal planes. Through the utilization of these landmarks and planes, the maxilla-based coordinate system provides a framework for accurately measuring and evaluating anatomical structures in the maxilla region

In order to prepare the data for measurement, we followed the method proposed in our previous research [15]. Initially, we used the Dolphin software (Version 11.8; Dolphin Imaging and Management Solutions; Chatsworth, CA, USA) to import the pre-treatment (T0) and post-treatment (T1) DICOM data. We performed voxel-based superimposition of the maxilla to align the maxillas of T0 and T1, ensuring that any changes in joint fossa position did not interfere (Figure 1). Afterwards, we reoriented and exported the T1 data as the T2 data. Next, we utilized the Mimics Research software (Version 19.0; Materialise, Leuven, Belgium) to import the T0, T1, and T2 data and reconstruct 3D models. We established a maxilla-based coordinate system in both the T0 and T1 models using four skeletal landmarks: ANS (the tip of the anterior nasal spine), PNS (the tip of the posterior nasal spine), OrL (the most inferior point of the left bony orbit), and OrR (the most inferior point of the right bony orbit) (Figure 2). As these four skeletal landmarks remain stable in adults, the coordinate systems of T0 and T1 were identical. Finally, we employed the T0 coordinate system to conduct three-dimensional measurements of the T0 temporomandibular joint structures, while the T1 coordinate system was used for three-dimensional measurements of the T2 (i.e., the reoriented T1) mandibular structures.

3D TMJ analysis

**Figure 3:**
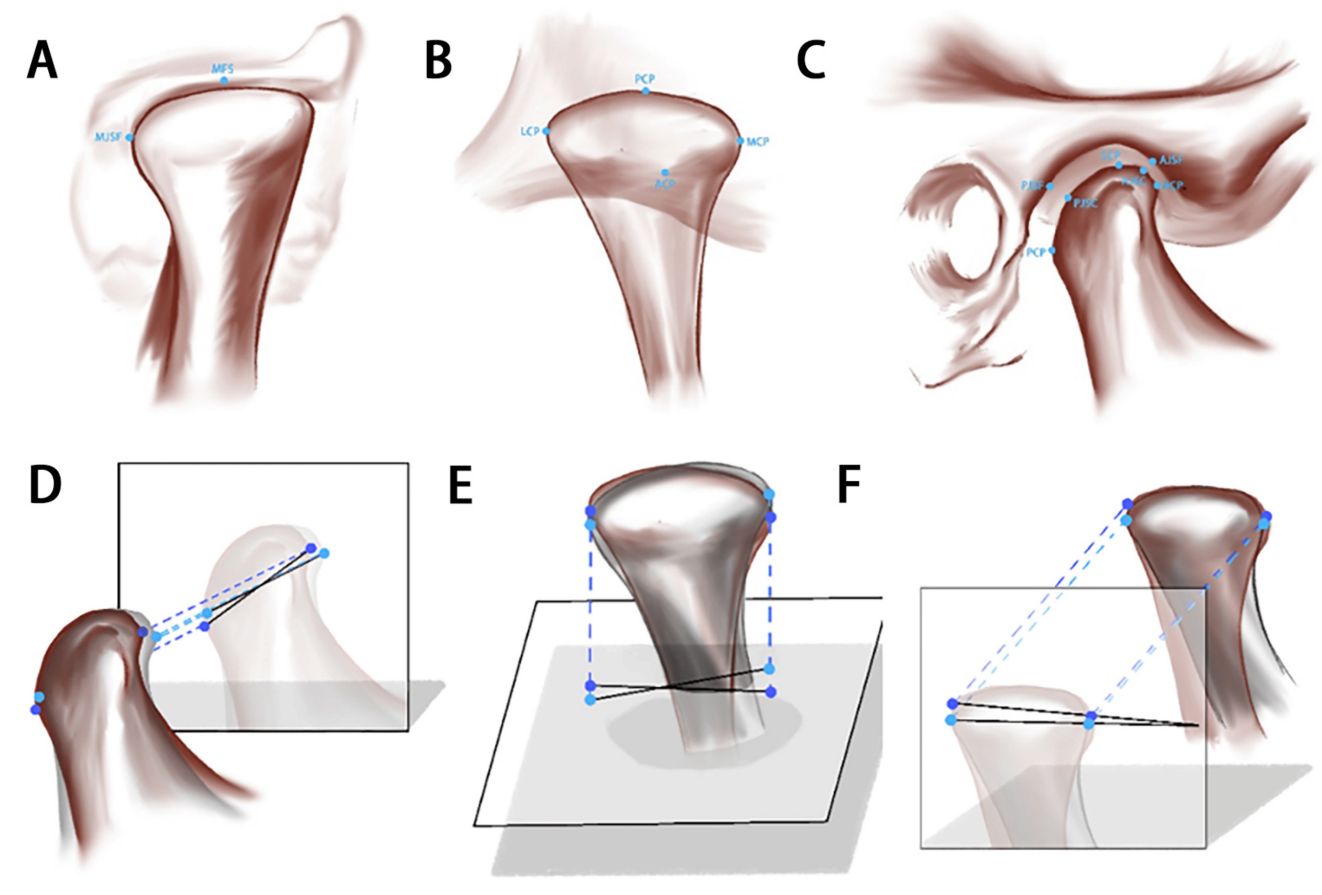
Temporomandibular joint anatomical landmarks for 3D analysis Temporomandibular joint anatomical landmarks for 3D analysis (A) Coronal plane landmarks (B) Horizontal plane landmarks (C) Sagittal plane landmarks (D) Condyle rotation projection in the sagittal plane (E) Condyle rotation projection in the horizontal plane (F) Condyle rotation projection in the coronal plane

We conducted a comprehensive analysis of the TMJ using a standardized and innovative 3D approach (Figure 3). Fifteen different landmarks were identified and digitized in a 3D volume (Table 1), which were then localized using a slice locator. We established 3D reference planes to facilitate measurements (Table 2).

**Table 1:** Definitions of facial and temporomandibular joint (TMJ) anatomical landmarks for 3D analysis

Landmark	Definition
Anterior nasal spine (ANS）	the tip of the anterior nasal spine
Posterior nasal spine (PNS）	the tip of the posterior nasal spine
Left bony orbit (OrL）	the most inferior point of the left bony orbit
Right bony orbit (OrR）	the most inferior point of the right bony orbit
Soft tissue mandibular fossa (MFS)	The most superior and mid-point of the soft tissue mandibular fossa region
Medial joint space “fossa point” (MJSf)	The most lateral point of the medial wall of mandibular fossa
Superior condylar point (SCP)	The most superior point of the condylar head
Lateral condylar point (LCP)	The most lateral point of the condylar head
Medial condylar point (MCP)	The most medial point of the condylar head
Anterior condylar point (ACP)	The most anterior point of the condylar head
Posterior condylar point (PCP)	The most posterior point of the condylar head
Anterior joint space “fossa point”(AJSf)	The most posterior point of the anterior wall of the mandibular fossa opposed to the shortest anterior condylar fossa distance
Anterior joint space “condylar point” (AJSc)	The most anterior point of the condyle opposed to the shortest anterior condylar fossa distance
Posterior joint space “fossa point” (PJSf)	The most anterior point of the posterior wall of the mandibular fossa opposed to the shortest posterior condylar fossa distance
Posterior joint space “condylar point” (PJSc)	The most posterior point of the condyle opposed to the shortest posterior condylar fossa distance

**Table 2:** Definitions of anatomical datum planes for 3D analysis ANS: the tip of the anterior nasal spine, PNS: the tip of the posterior nasal spine, OrL: the most inferior point of the left bony orbit, OrR: the most inferior point of the right bony orbit

Reference planes	Definition
Horizontal plane (HP)	The plane passing through ANS and PNS, running parallel to the OrL-OrR line
Sagittal plane (SP)	The plane passing through ANS and PNS while perpendicular to the horizontal plane
Frontal plane (FP)	The plane passing through ANS while perpendicular to both the sagittal plane and the horizontal plane

The measurements included various aspects of TMJ movement, such as anteroposterior, vertical, and mediolateral condylar rotations and positions. We also assessed the positions of the condyle within the joint and relative to the cranial base. Additionally, we measured the circumferential joint spaces (Table 3).

**Table 3:** Definitions of 3D measurements for volumetric images generated by cone-beam computed tomography (CBCT) SCP: Superior condylar point; ACP: Anterior condylar point; MCP: Medial condylar point; AJSc: Anterior joint space “condylar point”; AJSf: Anterior joint space “fossa point”; PJSf: Posterior joint space “fossa point”; PJSc: Posterior joint space “condylar point”; MFS: Soft tissue mandibular fossa; MJSf: Medial joint space “fossa point”; LCP: Lateral condylar point; PCP: Posterior condylar point; HP: Horizontal plane; SP: Sagittal plane; FP: Frontal plane

Measurement	Definition
Vertical point condylar position	The vertical distance between the SCP and HP
Anterior condylar point position	The vertical distance between the ACP and FP
Medial condylar point position	The vertical distance between the MCP and SP
Anterior joint space	The shortest distance between AJSc and AJSf
Posterior joint space	The shortest distance between PJSc and PJSf
Superior joint space	The shortest distance between SCP and MFS
Medial joint space	The shortest distance between MJSf and MCP
Condylar rotation in Horizontal plane	The angle between T0 and T2 MCP-LCP projected in the horizontal plane
Condylar rotation in Sagittal plane	The angle between T0 and T2 ACP-PCP projected in the sagittal plane
Condylar rotation in Frontal plane	The angle between T0 and T2 MCP-LCP projected in the frontal plane

In order to guarantee precision in evaluating changes in condylar position, we utilized two distinct approaches. The initial approach involved assessing condylar changes in relation to the craniofacial complex, using basal reference planes. The second approach involved applying a formula suggested by Pullinger and Hollender as a reference.

To evaluate the reliability of our measurements, we obtained CBCT measurements twice within a two-week period, and these measurements were performed by two different observers.

Overall, our study employed a systematic and innovative approach to analyze the TMJ in three dimensions. We utilized various landmarks, established reference planes, and employed multiple measurement methods to ensure accuracy and reliability in our findings.

Statistical analysis

The statistical analysis for this study utilized the SPSS software (Version 22.0; IBM, Armonk, NY, USA). In order to assess the level of agreement, both intra- and interobserver agreements were measured using the intraclass correlation coefficient (ICC). To examine the changes before and after treatment, a paired t-test and the Wilcoxon signed-rank test were employed.

## Results

A total of 88 samples from the TMJ were collected from 44 patients who had undergone extraction of their maxillary and mandibular bilateral premolars. The average time interval between the pre- and post-treatment records was 28 months. The reliability of all measurements, both within and between observers, was found to be good, with ICC values ranging from 0.800 to 0.995 (Table 4). Table 5 provides descriptive statistics of the linear and angular measurements taken before and after treatment.

**Table 4:** Intraclass correlation coefficient (ICC) for intra-observer and interobserver consistency

Measurement	Intra-­observer	Interobserver
Vertical point condylar position	0.995	0.989
Anterior condylar point position	0.932	0.954
Medial condylar point position	0.901	0.922
Condylar rotation in Frontal plane	0.885	0.836
Condylar rotation in Sagittal plane	0.943	0.880
Condylar rotation in Horizontal plane	0.932	0.830
Anterior joint space	0.940	0.959
Posterior joint space	0.964	0.963
Superior joint space	0.963	0.982
Medial joint space	0.911	0.904

**Table 5:** Mean values, standard deviations (SDs) and the results of analyses using paired t-tests and Wilcoxon signed-rank tests of the changes in mandibular condyle rotations and positions as well as joint spaces after treatment with therapeutic premolar extraction

Mandibular condyle measurements	Mean	SDs	p-value
Vertical point condylar position difference	-0.010	1.277	0.938
Anterior condylar point position difference	-0.413	0.736	＜0.001
Medial condylar point position difference	0.081	0.760	0.320
Condylar rotation in Frontal plane	0.621	2.080	0.006
Condylar rotation in Sagittal plane	0.567	4.150	0.204
Condylar rotation in Horizontal plane	-0.229	2.067	0.301
Anterior joint space difference	0.104	0.507	0.058
Posterior joint space difference	-0.324	0.695	＜0.001
Superior joint space difference	0.026	0.623	0.695
Medial joint space difference	-0.092	0.659	0.193

**Figure 4:**
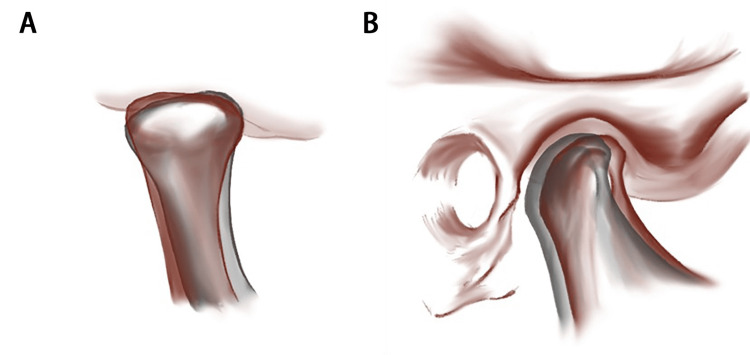
The translocation and rotation of condyles after orthodontic treatment involving four premolar extraction The translocation and rotation of condyles after orthodontic treatment involving four premolar extraction. The red figure represents the condition before orthodontic tooth extraction, while the green figure represents the condition after orthodontic tooth extraction. (A) The possible rotation of the condyle in the coronal plane (B) The possible movement of the condyles in the sagittal plane

In terms of positional changes, the study found a significant posterior displacement of the condyle in relation to the frontal plane (Moved back 0.41mm) following therapeutic extraction of the premolars (Figure 4). This resulted in a decrease in the posterior joint space (Reduced by 0.32mm). However, there were no statistically significant changes observed in the vertical or mediolateral translocation of the condyles and other joint spaces (Table 5).

Regarding rotational changes, the study found a significant medial elevation of the condyle in relation to the frontal plane (Rotated 0.62 degrees). However, no statistically significant changes were observed in the horizontal or sagittal rotation of the condyles.

## Discussion

TMJ is formed by bony structures (such as the condyle of the mandible and the glenoid fossa) and non-bony structures (such as the articular disc). In terms of measuring bony structures, CBCT measurements may be superior to MRI and can to some extent reflect the position of the intervertebral disc. In a study conducted by Gedlange et al. [16], the linear and angular measurements of CT and MRI were compared in order to identify various TMJ structures. The authors proposed that when there is a suspicion of a connection between TMDs and changes in bone density or fractures, the use of CT as a diagnostic tool should be considered. Additionally, previous research has also suggested that TMD patients who show clinical symptoms and have a potential risk of bone abnormalities in the TMJ should undergo additional evaluation with CBCT [17]. A review article has suggested that CBCT has become a cost-effective and dose-effective imaging modality for the diagnosis and assessment of various TMJ diseases. In the evaluation of TMJ, CBCT imaging has been found to be superior to traditional radiographic examinations and MRI [18]. The most important measurements related to the position of the condyle are condylar displacement and rotation, anterior joint space, posterior joint space, and superior joint space. Displacement and rotation of the condyle relative to the glenoid fossa may affect the tendency of disc displacement, occlusion, and vertical facial distance.

Regarding condylar displacement, we observed posterior displacement of the condyle during the treatment process. Our findings are similar to those of Alhammadi [14], but we observed less condylar displacement, which may be related to the extraction of the mandibular premolars. Some believe that during orthodontic treatment, the maxillary incisors have the effect of enclosing the mandible and causing it to move backward. However, the maxillary teeth and alveolar complex are unable to adapt by moving forward. As a result, the only option is to forcefully position the mandible in a backward position [19,20]. In a study by Wyatt [21], it was observed that when the maxillary anterior teeth move backward, the chewing muscles attempt to retract the mandible during mouth closure. This compensatory movement of the mandible can exert pressure on the condylar process, potentially leading to a posterior repositioning of the condylar process. We speculate that for patients who have had their two upper premolars extracted, the upper incisors retract while the lower incisors still maintain a lip-side tilt. This causes the upper dental-alveolar complex to push the lower jawbone backward, resulting in a greater distance of mandibular and condylar retrusion. However, for patients who have had all four premolars extracted, both the upper and lower incisors experience some degree of retraction, thus offsetting some of the compression on the lower jawbone and leading to less mandibular and condylar retrusion. Koide assumed that this may also be due to adaptive bone reconstruction of the temporomandibular joint during orthodontic treatment [12].

In contrast, several other studies have found that the position of the condyle, a part of the jaw joint, remains stable during orthodontic treatment. These studies have shown that there is no significant change in the condylar position when comparing cases with tooth extractions and cases without extractions [22-26]. However, Artun et al. [27] suggested that in some patients with a specific type of malocclusion, known as Angle Class II division 1, extracting certain teeth may result in a slightly more posterior condylar position. Nevertheless, this change was not found to be statistically significant. It is worth noting that most of these studies relied on cephalometric radiography or lateral cephalograms, which are two-dimensional imaging techniques. Due to the lack of strict calibration of the patient's head position during imaging, the obtained radiographic data was limited to measuring a segment between two points. These measurements neglected the influence of depth on the measurement values, thus providing only a partial understanding of the condylar position. The measured values are often smaller than those obtained using three-dimensional spatial measurement methods. In this study, not only were the pre- and post-treatment head positions of the patients calibrated and overlapped, but the three-dimensional levels of these joint spaces were detected, and a new measurement coordinate system was added to accurately measure condylar displacement or rotation in a three-dimensional overlapping manner.

Multiple studies using MRI have investigated the correlation between joint space and posterior condylar position as well as intervertebral disc position. The findings indicate that disc displacement joints often exhibit posterior condylar displacement, and the position of the condyle can serve as an indicator of anterior disc displacement [28,29]. Ikeda and Kawamura [30,31] utilized CBCT and MRI to demonstrate that intervertebral disc displacement in adolescents and young individuals can result in changes in condyle position within the fossa. The extent of these changes in joint space depends on the direction and severity of the disc displacement. In cases of reducible disc displacement, the condyle moves posteriorly within the fossa, leading to an increase in anterior space and a decrease in posterior space. Conversely, non-reducible disc displacement causes posterior movement of the condyle, resulting in a slight decrease in upper joint space and an increase in medial joint space due to displacement in the anterior-posterior direction. Alhammadi proposed that significant posterior condylar positioning may cause excessive stretching of the lateral pterygoid muscle. Since this muscle is connected to the temporomandibular joint and disc, subsequent splinting or spasms of these muscles can potentially contribute to varying degrees of disc displacement [14]. Therefore, for patients undergoing orthodontic treatment with tooth extraction, attention may need to be paid to maintaining the stability of the temporomandibular joint during orthodontic treatment.

Regarding condylar rotation, statistically significant elevation of the long axis of the condyle on the medial side in the frontal projection indicates a slight rotation of the mandibular ramus posteriorly [32]. Patients undergoing orthodontic treatment with premolar extractions face the same risk of increased vertical dimension as those undergoing non-extraction orthodontic treatment [33-35]. Combining simulation experiments on skull models and the experience of temporomandibular joint doctors, this rotational change in the frontal projection of the condyle may be related to the increased vertical dimension after tooth extraction orthodontics. At the same time, the rotational change of the condyle may also lead to spontaneous resorption of the condyle [36-38], which needs to be given sufficient attention.

Statistically, patients undergoing orthodontic treatment do not have a higher or lower risk of TMD [39,40]. However, this posterior displacement of the condyle of approximately 0.4 mm should be evaluated in the long term, especially when this effect is related to the possibility of TMD occurrence, it has clinical significance. The potential impact of premolar extraction and mandibular incisor retraction on the posterior condylar position depends on the initial position of the condyle and whether the patient has predisposing factors for TMD before orthodontic treatment. For patients with anterior condylar positioning before treatment, a more stable condylar fossa relationship may be achieved after treatment, while for patients with intermediate or posterior condylar positioning before treatment, there may be an increased risk of condylar protrusion beyond the physiological position after treatment. However, to determine whether this effect is temporary or long-term, extensive radiological research and long-term clinical follow-up are still needed.

This study has several strengths. First, it innovatively studied the rotation of the condyle, which was difficult to achieve in previous studies based on two-dimensional imaging data. Second, compared with other similar studies with sample sizes of over 20, the increased sample size in our study may enhance the dependability of the findings. Furthermore, the utilization of a consistent upper jaw-based coordinate system and voxel-based superimposition technique in data preparation eliminates the potential influence of head position variations during CBCT imaging. As a result, we are able to precisely identify landmarks and conduct three-dimensional measurements.

There are two limitations to this study. First, only the imaging data at the end of orthodontic treatment was measured, and long-term follow-up observation was not conducted, so it is impossible to determine whether the changes in the temporomandibular joint are temporary or long-term. Long-term follow-up of patients is still needed to confirm whether the changes in condylar position are temporary. Second, MRI was not combined in our study, so we cannot accurately evaluate the changes in intervertebral disc position and morphology.

Our study revealed the tendency for posterior displacement and rotation of the condyle after premolar extraction in adults. This has clinical significance for orthodontic treatment. It allows orthodontists to anticipate the changes in the temporomandibular joint that may accompany orthodontic treatment in a more intuitive way. Understanding the condylar motion and amount of motion after orthodontic treatment with premolar extraction can help maximize the understanding of temporomandibular joint movement and tailor orthodontic treatment plans for different patients, thereby controlling the temporomandibular joint to be in a good physiological state and benefiting future patients.

## Conclusions

This study examined the alterations in the TMJ among adults undergoing orthodontic treatment involving the extraction of four premolars. Gaining insight into the precise changes occurring in the TMJ during orthodontic treatment can provide a valuable framework for devising treatment strategies and ensuring the stability of the TMJ in patients. Nonetheless, further research is required to investigate the long-term clinical implications of these changes through follow-up studies.
